# The genome sequence of a bluebottle, *Calliphora vomitoria* (Linnaeus, 1758)

**DOI:** 10.12688/wellcomeopenres.18891.1

**Published:** 2023-02-21

**Authors:** Olga Sivell, Natural History Museum Genome Acquisition Lab, Darwin Tree of Life Barcoding collective, Wellcome Sanger Institute Tree of Life programme, Wellcome Sanger Institute Scientific Operations: DNA Pipelines collective, Tree of Life Core Informatics collective, Darwin Tree of Life Consortium

**Affiliations:** 1 Natural History Museum, London, UK

**Keywords:** Calliphora vomitoria, bluebottle, blowfly, genome sequence, chromosomal, Diptera

## Abstract

We present a genome assembly from an individual male *Calliphora vomitoria* (a bluebottle; Arthropoda; Insecta; Diptera; Calliphoridae). The genome sequence is 708 megabases in span. Most of the assembly is scaffolded into six chromosomal pseudomolecules, including the assembled X sex chromosome. The mitochondrial genome has also been assembled and is 16.2 kilobases in length. Gene annotation of this assembly on Ensembl identified 12,917 protein coding genes.

## Species taxonomy

Eukaryota; Metazoa; Ecdysozoa; Arthropoda; Hexapoda; Insecta; Pterygota; Neoptera; Endopterygota; Diptera; Brachycera; Muscomorpha; Oestroidea; Calliphoridae; Calliphorinae; *Calliphora; Calliphora vomitoria* (Linnaeus, 1758) (NCBI:txid27454).

## Background

*Calliphora vomitoria* belongs in the Diptera family Calliphoridae (blowflies). The species from the genus *Calliphora* are commonly called bluebottles due to their shiny metallic appearance. *Calliphora vomitoria* is easily identifiable due to the orange hairs on the postgena and lower part of gena (a ‘ginger beard’) that contrast with the black base colour (covered with grey dusting) on those parts of the head. The lower calypters are brown with a white rim and with dark brown hairs on the upper side. The anterior thoracic spiracle and basicosta are dark brown to black ( Rognes, 1991; Sivell, 2021).

This species is common and widely distributed in Britain ( Davies, 1990; Davies, 1999; Davies & Laurence, 1992; MacLeod, 1943, MacLeod, 1963; MacLeod & Donnelly, 1956; Sivell, 2021); although less synanthropic than *C. vicina* ( Draber-Mońko, 2004; Hwang & Turner, 2005). It prefers shady sites and is common in woodland ( Davies, 1999; Green, 1951; MacLeod & Donnelly, 1956; van Emden, 1954) and uplands ( Davies, 1990), up to 1070 m ( Sivell, 2021), also in gardens and parks. The flight period is from March to October/November, although adults are occasionally seen during winter on warmer days ( Sivell, 2021). The adults are attracted to carrion, faeces, stinkhorn fungus ( *Phallus impudicus* Linnaeus, 1753), flowering plants and ripe fruit; they feed mainly on sugar.

*Calliphora vomitoria* is oviparous and anautogenous: females require a protein meal to reach maturity and produce eggs ( Rivers & Dahlem, 2014). The larvae are saprophagous and feed on carrion. A female fly oviposits on the carcass, choosing shaded and moist locations (e.g. the mouth, ears, eyes, anus) to avoid desiccation of the eggs. This species has a strong preference for large carrion such as sheep ( Davies, 1990, Davies, 1999) or human bodies, making it potentially useful in forensics. Its developmental rates have been researched using populations from North America ( Greenberg & Tantawi, 1993; Kamal, 1958) and Europe ( Marchenko, 2001; Niederegger *et al*., 2010; Niederegger *et al.*, 2013) including Britain ( Davies & Ratcliffe, 1994). *Calliphora vomitoria* can also cause secondary myiasis in living animals which have a primary infestation from another species. This is most frequent in sheep and is referred to as ‘sheep-strike’ ( Haddow & Thomson, 1937; MacLeod, 1943; Morris & Titchener, 1997; Taylor *et al.*, 2013). *Calliphora vomitoria* was also reported from a case of orbital myiasis in a human in USA ( Wood & Slight, 1970).

## Genome sequence report

**Figure 1.**
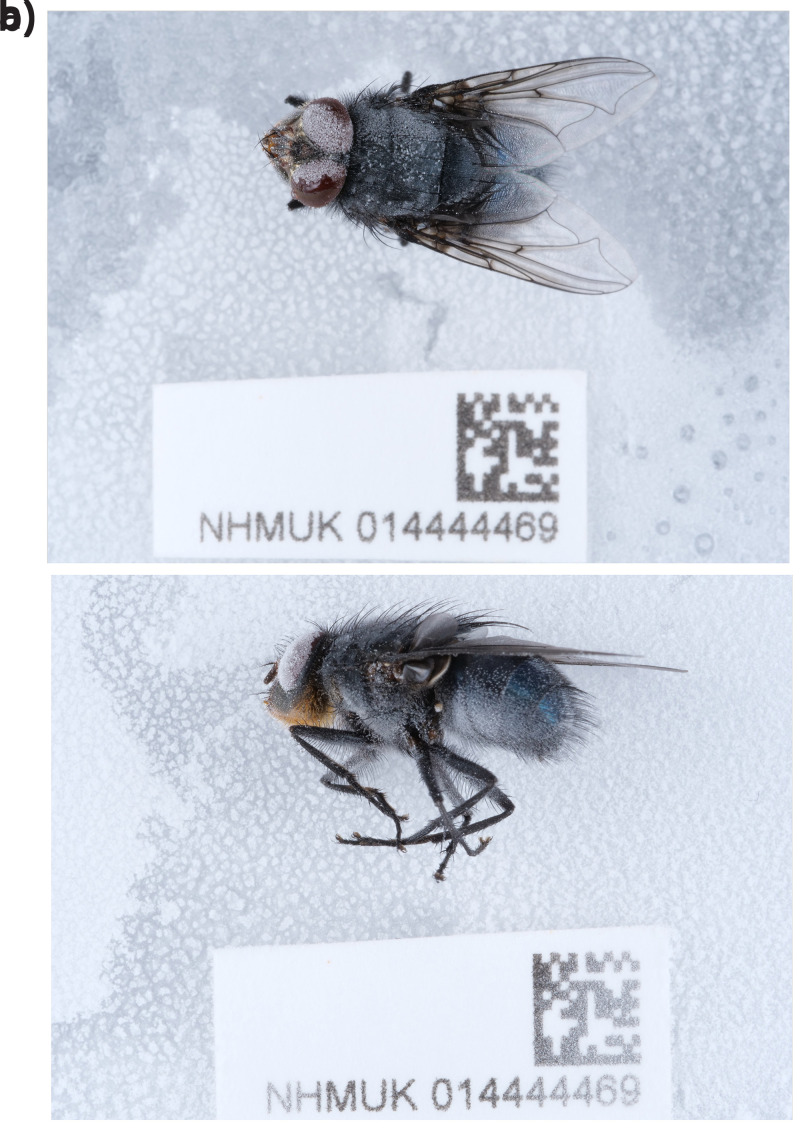
*Calliphora vomitoria* (Linnaeus, 1758) specimen NHMUK014444469. Photographs taken during sample preservation and processing. **a**) The specimen in dorsal view. **b**) The specimen in lateral view. Photographs by O. Sivell. © Trustees of the Natural History Museum, London.

The genome was sequenced from one male *Calliphora vomitoria* specimen ( Figure 1) collected from Hever Castle, UK (51.188, 0.12). A total of 54-fold coverage in Pacific Biosciences single-molecule HiFi long reads was generated. Primary assembly contigs were scaffolded with chromosome conformation Hi-C data. Manual assembly curation corrected 36 missing joins or mis-joins and removed five haplotypic duplications, reducing the assembly length by 0.53% and the scaffold number by 8.05%, and increasing the scaffold N50 by 1.38%.

**Figure 2.**
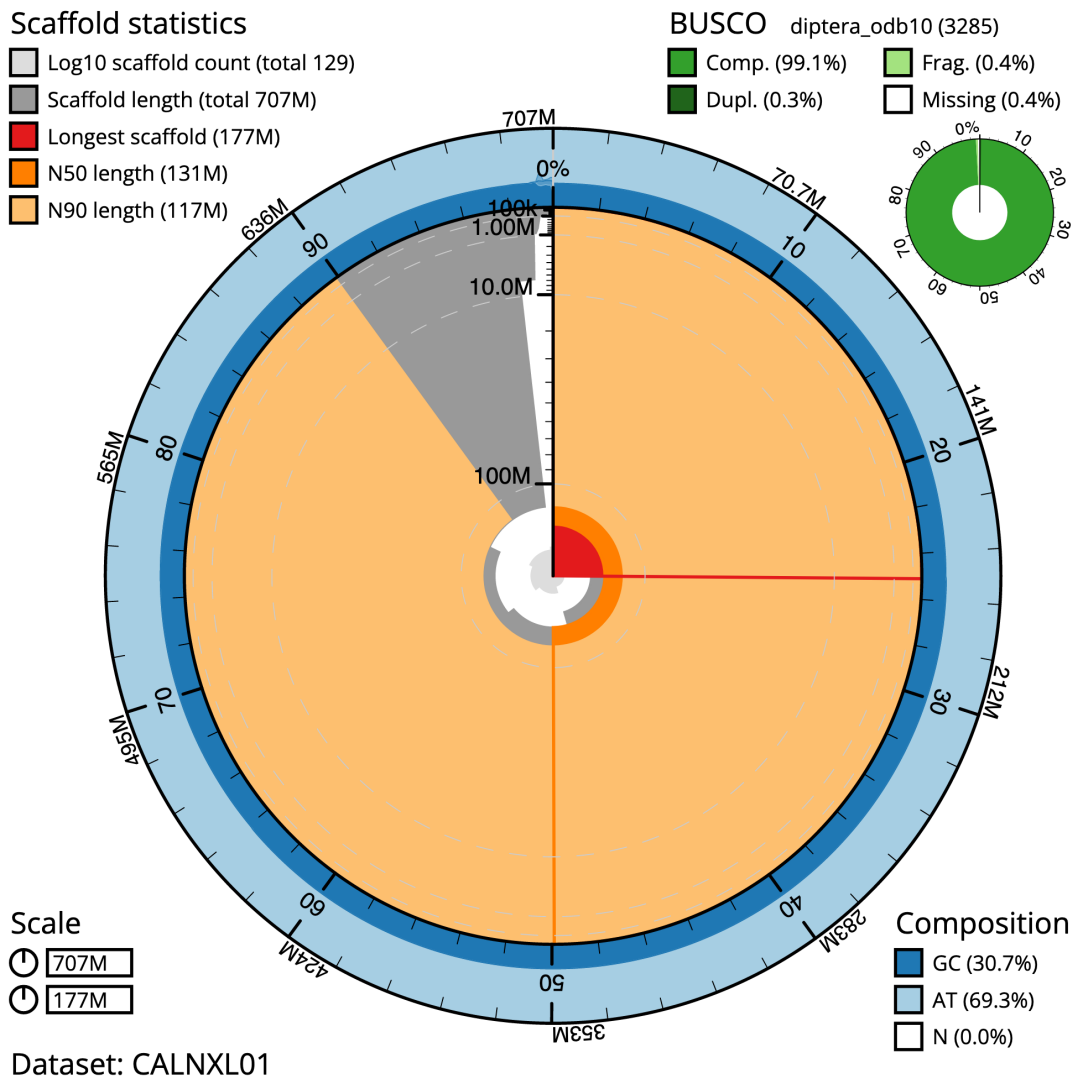
Genome assembly of *Calliphora vomitoria*, idCalVomi1.2: metrics. The BlobToolKit Snailplot shows N50 metrics and BUSCO gene completeness. The main plot is divided into 1,000 size-ordered bins around the circumference with each bin representing 0.1% of the 706,678,423 bp assembly. The distribution of scaffold lengths is shown in dark grey with the plot radius scaled to the longest scaffold present in the assembly (177,447,591 bp, shown in red). Orange and pale-orange arcs show the N50 and N90 sequence lengths (131,076,759 and 116,750,479 bp), respectively. The pale grey spiral shows the cumulative scaffold count on a log scale with white scale lines showing successive orders of magnitude. The blue and pale-blue area around the outside of the plot shows the distribution of GC, AT and N percentages in the same bins as the inner plot. A summary of complete, fragmented, duplicated and missing BUSCO genes in the diptera_odb10 set is shown in the top right. An interactive version of this figure is available at https://blobtoolkit.genomehubs.org/view/idCalVomi1.1/dataset/CALNXL01/snail.

The final assembly has a total length of 707.6 Mb in 137 sequence scaffolds with a scaffold N50 of 130.6 Mb ( Table 1). Most (99.04%) of the assembly sequence was assigned to six chromosomal-level scaffolds, representing five autosomes and the X sex chromosome. Chromosome-scale scaffolds confirmed by the Hi-C data are named in order of size ( Figure 2– Figure 5; Table 2). The assembly has a BUSCO v5.3.2 ( Manni *et al.*, 2021) completeness of 99.1% using the OrthoDB v10 Diptera reference set ( *n* = 3,285). While not fully phased, the assembly deposited is of one haplotype. Contigs corresponding to the second haplotype have also been deposited.

**Table 1.** Genome data for *Calliphora vomitoria*, idCalVomi1.2.

Project accession data		
Assembly identifier	idCalVomi1.2	
Species	*Calliphora vomitoria*	
Specimen	idCalVomi1	
NCBI taxonomy ID	27454	
BioProject	PRJEB52479	
BioSample ID	SAMEA8534291e	
Isolate information	idCalVomi1 thorax: PacBio	
Assembly metrics *		*Benchmark*
Consensus quality (QV)	59.2	*≥ 50*
*k*-mer completeness	100%	*≥ 95%*
BUSCO **	C:99.1%[S:98.8%,D:0.3%], F:0.4%,M:0.4%,n:3,285	*C ≥ 95%*
Percentage of assembly mapped to chromosomes	99.04%	*≥ 95%*
Sex chromosomes	X chromosome assembled	*localised homologous pairs*
Organelles	Mitochondrial genome assembled	*complete single alleles*
Raw data accessions		
PacificBiosciences SEQUEL II	ERR9682745, ERR9682746	
Hi-C Illumina	ERR9682483	
Genome assembly		
Assembly accession	GCA_942486065.2	
*Accession of alternate haplotype*	GCA_942486075.2	
Span (Mb)	707.6	
Number of contigs	301	
Contig N50 length (Mb)	14.6	
Number of scaffolds	137	
Scaffold N50 length (Mb)	130.6	
Longest scaffold (Mb)	178.0	
Genome annotation		
Number of protein-coding genes	12,917	
Non-coding genes	2,327	
Gene transcripts	20,875	

* Assembly metric benchmarks are adapted from column VGP-2020 of “Table 1: Proposed standards and metrics for defining genome assembly quality” from ( Rhie *et al.*, 2021). ** BUSCO scores based on the diptera_odb10 BUSCO set using version 5.3.2. C = complete [S = single copy, D = duplicated], F = fragmented, M = missing, n = number of orthologues in comparison. A full set of BUSCO scores is available at https://blobtoolkit.genomehubs.org/view/idCalVomi1.1/dataset/CALNXL01/busco.

**Figure 3.**
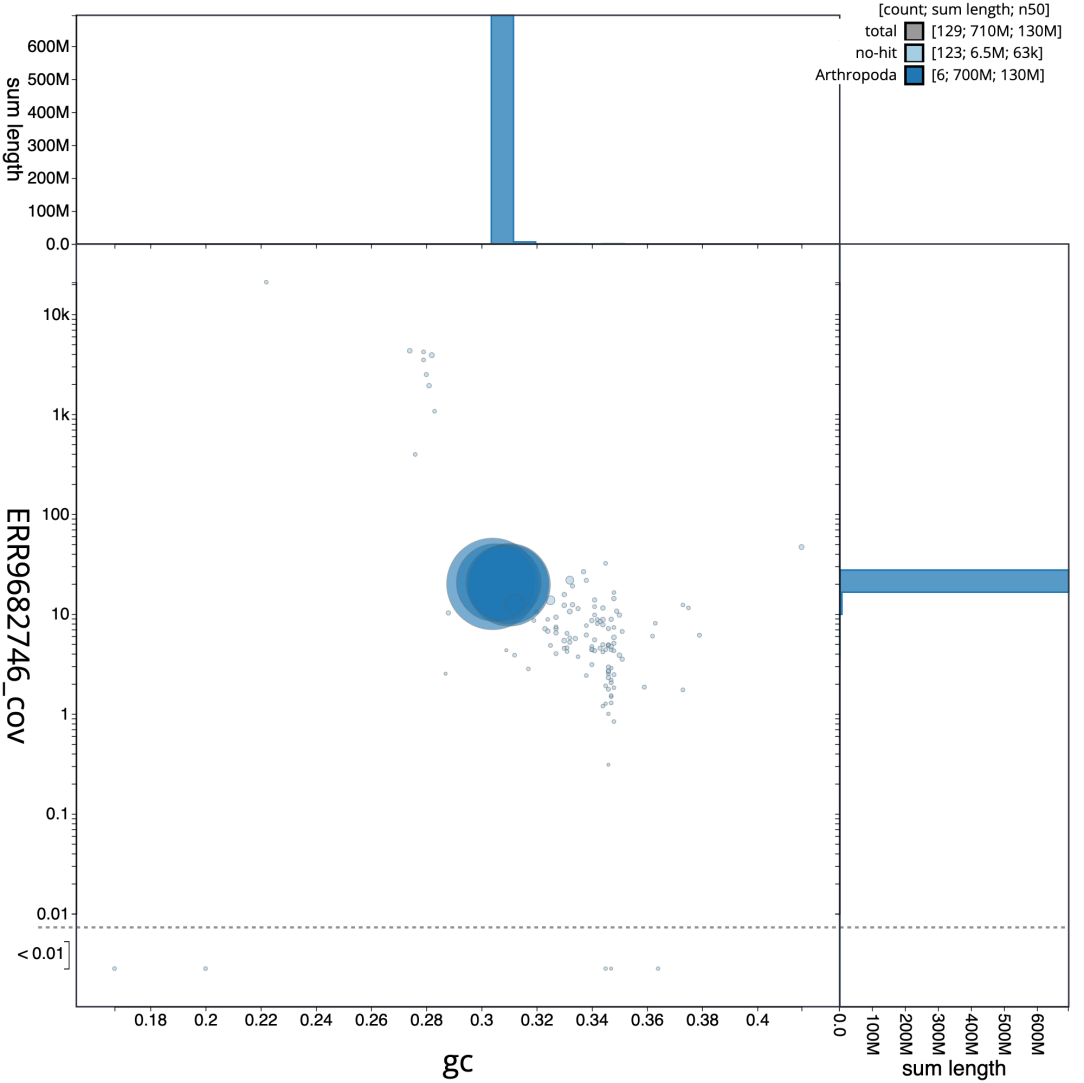
Genome assembly of *Calliphora vomitoria*, idCalVomi1.2: GC coverage. BlobToolKit GC-coverage plot. Scaffolds are coloured by phylum. Circles are sized in proportion to scaffold length. Histograms show the distribution of scaffold length sum along each axis. An interactive version of this figure is available at https://blobtoolkit.genomehubs.org/view/idCalVomi1.1/dataset/CALNXL01/blob.

**Figure 4.**
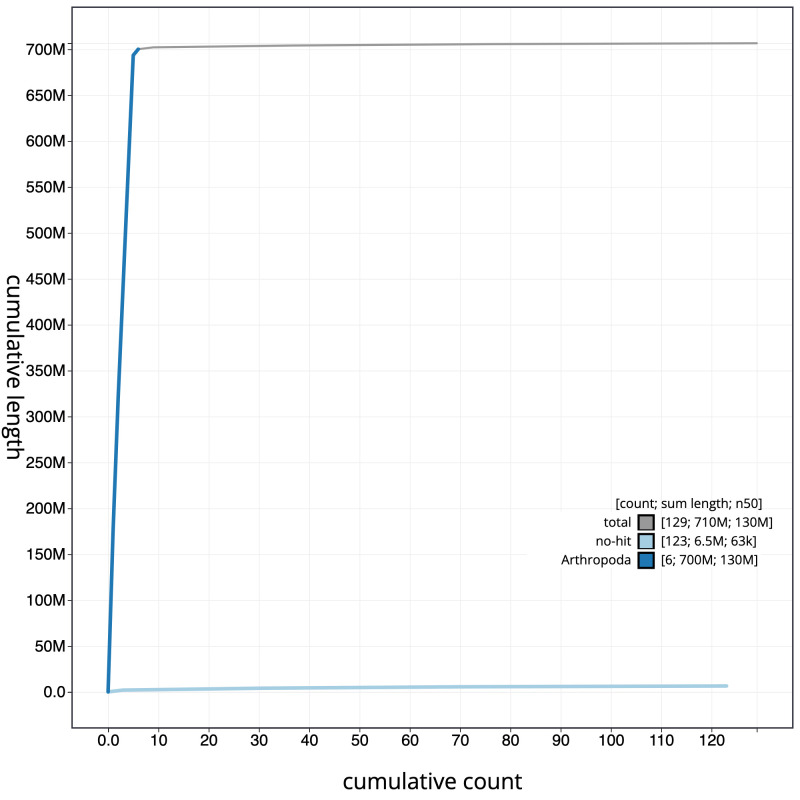
Genome assembly of *Calliphora vomitoria*, idCalVomi1.2: cumulative sequence. BlobToolKit cumulative sequence plot. The grey line shows cumulative length for all scaffolds. Coloured lines show cumulative lengths of scaffolds assigned to each phylum using the buscogenes taxrule. An interactive version of this figure is available at https://blobtoolkit.genomehubs.org/view/idCalVomi1.1/dataset/CALNXL01/cumulative.

**Figure 5.**
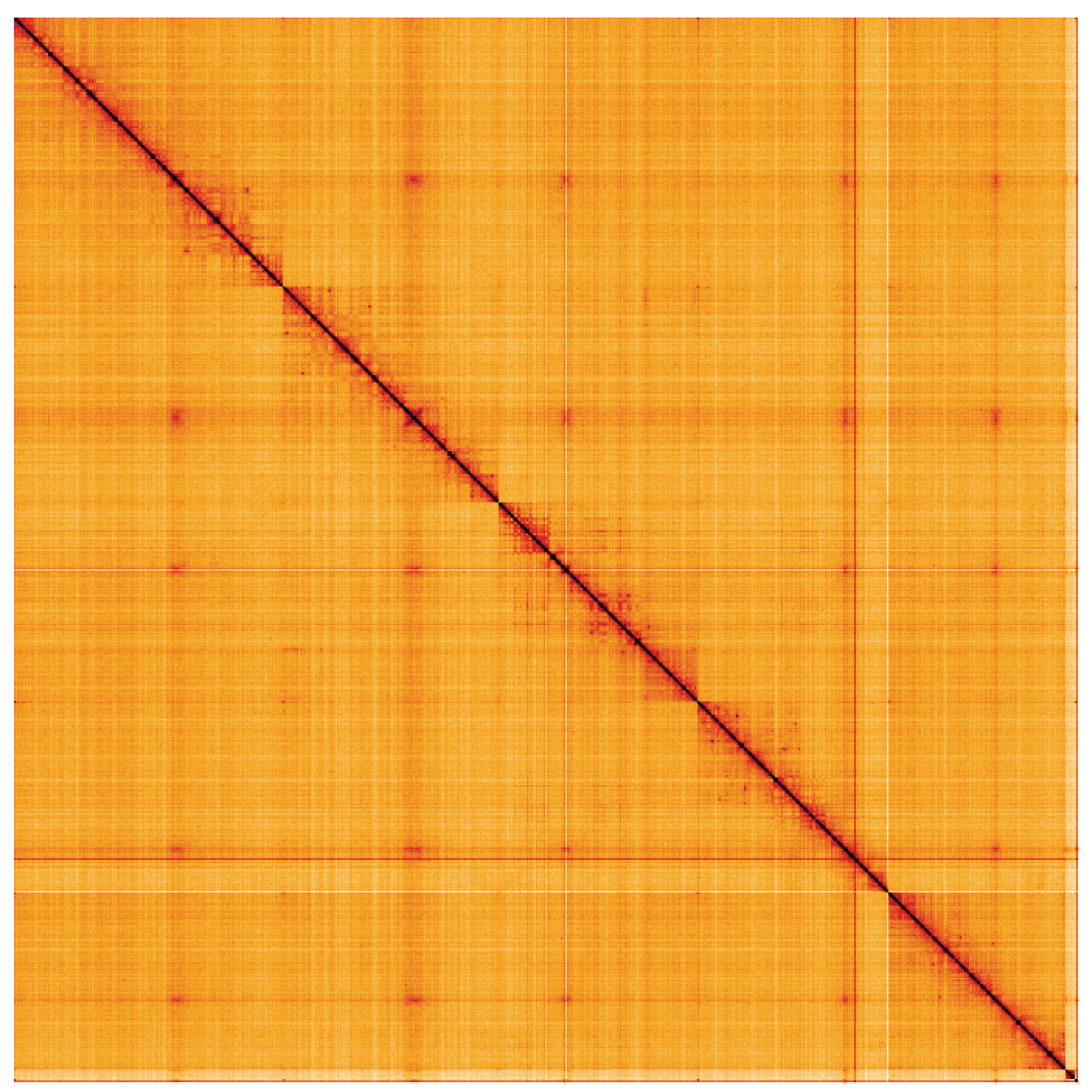
Genome assembly of *Calliphora vomitoria*, idCalVomi1.2: Hi-C contact map. Hi-C contact map of the idCalVomi1.2 assembly, visualised using HiGlass. Chromosomes are shown in order of size from left to right and top to bottom. An interactive version of this figure may be viewed at https://genome-note-higlass.tol.sanger.ac.uk/l/?d=P7Xf5smoSJG-hLdBKd_gtw.

**Table 2.** Chromosomal pseudomolecules in the genome assembly of *Calliphora vomitoria*, idCalVomi1.

INSDC accession	Chromosome	Size (Mb)	GC (%)
OW818029.2	1	178.04	30
OW818030.2	2	143.36	31
OW818031.2	3	130.62	30.5
OW818032.2	4	124.48	30
OW818033.2	5	117.30	30.5
OW818034.2	X	7.05	31
OW818035.2	MT	0.02	22

## Genome annotation report

The *C. vomitoria* (GCA_942486065.1) genome assembly was annotated using the Ensembl rapid annotation pipeline ( Table 1; https://rapid.ensembl.org/Calliphora_vomitoria_GCA_942486065.1/). The resulting annotation includes 20,875 transcribed mRNAs from 12,917 protein-coding and 2,327 non-coding genes.

## Methods

### Sample acquisition and nucleic acid extraction

A male *Calliphora vomitoria* (idCalVomi1) was collected from Hever Castle (latitude 51.188, longitude 0.12) on 27 August 2020. The specimen was caught by Olga Sivell (Natural History Museum) using an aerial net. The specimen was also identified by Olga Sivell and preserved on dry ice.

DNA was extracted at the Tree of Life laboratory, Wellcome Sanger Institute (WSI). The idCalVomi1 sample was weighed and dissected on dry ice with tissue set aside for Hi-C sequencing. Thorax tissue was disrupted using a Nippi Powermasher fitted with a BioMasher pestle. High molecular weight (HMW) DNA was extracted using the Qiagen MagAttract HMW DNA extraction kit. HMW DNA was sheared into an average fragment size of 12–20 kb in a Megaruptor 3 system with speed setting 30. Sheared DNA was purified by solid-phase reversible immobilisation using AMPure PB beads with a 1.8X ratio of beads to sample to remove the shorter fragments and concentrate the DNA sample. The concentration of the sheared and purified DNA was assessed using a Nanodrop spectrophotometer and Qubit Fluorometer and Qubit dsDNA High Sensitivity Assay kit. Fragment size distribution was evaluated by running the sample on the FemtoPulse system.

### Sequencing

Pacific Biosciences HiFi circular consensus and 10X Genomics read cloud DNA sequencing libraries were constructed according to the manufacturers’ instructions. DNA sequencing was performed by the Scientific Operations core at the WSI on the Pacific Biosciences SEQUEL II (HiFi) instrument. Hi-C data were also generated from head tissue of idCalVomi1 using the Arima v2 kit and sequenced on the Illumina NovaSeq 6000 instrument.

### Genome assembly

Assembly was carried out with Hifiasm ( Cheng *et al.*, 2021) and haplotypic duplication was identified and removed with purge_dups ( Guan *et al.*, 2020). The assembly was scaffolded with Hi-C data ( Rao *et al.*, 2014) using YaHS ( Zhou *et al.*, 2022), The assembly was checked for contamination as described previously ( Howe *et al.*, 2021). Manual curation ( Howe *et al.*, 2021) was performed using HiGlass ( Kerpedjiev *et al.*, 2018) and Pretext ( Harry, 2022). The mitochondrial genome was assembled using MitoHiFi ( Uliano-Silva *et al.*, 2022), which performed annotation using MitoFinder ( Allio *et al.*, 2020). The genome was analysed and BUSCO scores generated within the BlobToolKit environment ( Challis *et al.*, 2020). Table 3 contains a list of all software tool versions used, where appropriate.

**Table 3.** Software tools and versions used.

Software tool	Version	Source
BlobToolKit	3.4.0	Challis *et al.*, 2020
Hifiasm	0.16.1-r375	Cheng *et al.*, 2021
HiGlass	1.11.6	Kerpedjiev *et al.*, 2018
MitoHiFi	1	Uliano-Silva *et al.*, 2022
PretextView	0.2	Harry, 2022
purge_dups	1.2.3	Guan *et al.*, 2020
YaHS	yahs-1.1.91eebc2	Zhou *et al*., 2022

### Genome annotation

The Ensembl gene annotation system ( Aken *et al.*, 2016) was used to generate annotation for the *Calliphora vomitoria* assembly (GCA_942486065.1). Annotation was created primarily through alignment of transcriptomic data to the genome, with gap filling via protein to-genome alignments of a select set of proteins from UniProt ( UniProt Consortium, 2019).

### Ethics/compliance issues

The materials that have contributed to this genome note have been supplied by a Darwin Tree of Life Partner. The submission of materials by a Darwin Tree of Life Partner is subject to the Darwin Tree of Life Project Sampling Code of Practice. By agreeing with and signing up to the Sampling Code of Practice, the Darwin Tree of Life Partner agrees they will meet the legal and ethical requirements and standards set out within this document in respect of all samples acquired for, and supplied to, the Darwin Tree of Life Project. Each transfer of samples is further undertaken according to a Research Collaboration Agreement or Material Transfer Agreement entered into by the Darwin Tree of Life Partner, Genome Research Limited (operating as the Wellcome Sanger Institute), and in some circumstances other Darwin Tree of Life collaborators.

## Data Availability

European Nucleotide Archive: *Calliphora vomitoria*. Accession number PRJEB52479; https://identifiers.org/ena.embl/PRJEB52479 ( Wellcome Sanger Institute, 2022). The genome sequence is released openly for reuse. The *Calliphora vomitoria* genome sequencing initiative is part of the Darwin Tree of Life (DToL) project. All raw sequence data and the assembly have been deposited in INSDC databases. Raw data and assembly accession identifiers are reported in Table 1.

## Author information

Members of the Natural History Museum Genome Acquisition Lab are listed here: https://doi.org/10.5281/zenodo.4790043.

Members of the Darwin Tree of Life Barcoding collective are listed here: https://doi.org/10.5281/zenodo.4893703.

Members of the Wellcome Sanger Institute Tree of Life programme are listed here: https://doi.org/10.5281/zenodo.4783585.

Members of Wellcome Sanger Institute Scientific Operations: DNA Pipelines collective are listed here: https://doi.org/10.5281/zenodo.4790455.

Members of the Tree of Life Core Informatics collective are listed here: https://doi.org/10.5281/zenodo.5013541.

Members of the Darwin Tree of Life Consortium are listed here: https://doi.org/10.5281/zenodo.4783558.
