# Supplementary material for: The genome sequence of a bluebottle, Calliphora vomitoria (Linnaeus, 1758)
Source: Wellcome Open Res. 2023 Feb 21;8:93. [Version 1] doi: 10.12688/wellcomeopenres.18891.1 (PMC10762291; doi:10.12688/wellcomeopenres.18891.1)
Supplement: Peer review history

# Reviewer report: Reviewer response for version 1

**DOI:** 10.21956/wellcomeopenres.20947.r70522

**Published:** 2024-01-02

**Authors:** Xin Liu

**Affiliations:** 1 State Key Laboratory of Agricultural Genomics, BGI (Beijing Genomics Institute)-Shenzhen, Shenzhen, China

Dr. Olga Sivell present a genome assembly from an individual male Calliphora vomitoria, which is an important genome data for this species study. There are some points should be revised.

1. The author has employed hic data to assemble the genome at the chromosome level and has assigned chromosome numbers based on their lengths, considering the shortest chromosome as the X chromosome. However, it is recommended to provide additional evidences, such as marker genes of X chromosome or collinearity with other model species, to support the claim that the shortest chromosome indeed corresponds to the X chromosome.
2. The manuscript includes data from PacBio HiFi CCS, 10x genomics, and HiC libraries, but lacks an explanation of how the data from 10x genomics was utilized in the assembly method. It is advised to provide a detailed description, possibly in the form of a flow chart, that outlines the software, version, and parameters employed in each step of the assembly process.
3. The YaHC tool, which the author used for assembling chromosomes, requires an update since the article has been published in Bioinformatics, while the author cited the reference from bioRxiv. Furthermore, the heat map in Figure 5 should clearly mark the boundary points of each chromosome as it is currently unclear.
4. In the abstract, the author mentions the assembly of the mitochondria; however, there is a lack of description regarding the mitochondrial genome and gene annotation in the Results. It is recommended to address this gap and provide the necessary details.
5. The general genome annotation should include the annotation of repeat sequences. Therefore, it is suggested that the author supplements the annotation results and provides a description of the methodology employed for annotating repeat sequences.

Are sufficient details of methods and materials provided to allow replication by others?

Yes

Is the rationale for creating the dataset(s) clearly described?

Yes

Are the datasets clearly presented in a useable and accessible format?

Yes

Are the protocols appropriate and is the work technically sound?

Yes

Reviewer Expertise:

plant genomics

I confirm that I have read this submission and believe that I have an appropriate level of expertise to confirm that it is of an acceptable scientific standard.

# Reviewer report: Reviewer response for version 1

**DOI:** 10.21956/wellcomeopenres.20947.r58999

**Published:** 2023-06-19

**Authors:** Maxwell J Scott

**Affiliations:** 1 North Carolina State University, Raleigh, USA

The authors report a very high-quality assembly of the genome of the blow fly, *Calliphora vomitoria*. I have a few minor comments for the authors to consider:

- Since this is a genome paper, some information in the Background on the expected number of chromosomes and genome size if known (or genome sizes for other flies in the genus). Most blow flies have five autosomes and X and Y sex chromosomes. There may not be any cytogenetic studies published for this species but other *Calliphora* species have been studied.
- In the methods 10XGeneomics libraries were made in addition to the PacBio. However, it is not clear from the methods or results how the 10X reads contributed to the final assembly.
- How was the X chromosome scaffold identified? Since DNA was only sequenced from a single male, I assume this was done by comparison to other blow fly genomes.
- Some additional labels on Figure 2 would help understand what is quite a complex figure.
- I think the X chromosome scaffold is the small square at the bottom right of Figure 5. If so, a label and arrow pointing to this part of the plot would be helpful.

Are sufficient details of methods and materials provided to allow replication by others?

Yes

Is the rationale for creating the dataset(s) clearly described?

Yes

Are the datasets clearly presented in a useable and accessible format?

Yes

Are the protocols appropriate and is the work technically sound?

Yes

Reviewer Expertise:

Insect genetics

I confirm that I have read this submission and believe that I have an appropriate level of expertise to confirm that it is of an acceptable scientific standard.
